# Molecular Detection of Porcine Torovirus in Piglets with Diarrhea in Southwest China

**DOI:** 10.1155/2013/984282

**Published:** 2013-12-26

**Authors:** Yuancheng Zhou, Lei Chen, Ling Zhu, Zhiwen Xu

**Affiliations:** ^1^Animal Biotechnology Center, College of Veterinary Medicine of Sichuan Agricultural University, Ya'an 625014, China; ^2^Key Laboratory of Animal Disease and Human Health of Sichuan Province & Animal Biotechnology Center, College of Veterinary Medicine of Sichuan Agricultural University, Ya'an 625014, China

## Abstract

Porcine torovirus (PToV) was detected from intestinal samples of piglets with diarrhea from 20 farms in southwest China. The total prevalence of PToV was 45% (9 out of 20 farms); it was the first detection of PToV in China, and also the study analyzed the phylogenetic relationships between the Chinese PToV and PToV reference strains as well as other representative toroviruses. Genetic and phylogenetic analysis showed the existence of genetic diversity among geographically separated PToV. Statistical analysis of the PToV positive rate as well as a survey for other enteric pathogens in diarrheic pigs suggests that PToV may play a role as a causative agent of severe diarrhea in piglets.

## 1. Introduction

*Toroviruses* are enveloped, positive-stranded polyadenylated RNA viruses, which belong to the family *Coronaviridae* and also toroviruses are potential gastroenteritis causing agents affecting humans, calves, pigs, and horses [1–6]. In 1982, bovine torovirus (BToV) was first isolated from a case of neonatal calf diarrhea in the United States and BToV was reported to be related to calf diarrhea in experimentally infected gnotobiotic calves and under field conditions. Porcine torovirus is a member of the genus *Torovirus *(family Coronaviridae, order Nidovirales), and its genome organization is similar to other toroviruses, consisting of ~28000 nucleotides organized into five ORFs expressing a replicase polyprotein and four structural proteins: spike (S), membrane (M), hemagglutinin-esterase (HE) and nucleocapsid (N) [6–8]. Porcine torovirus has been reported in Canada, South Africa and European countries, Italy, Hungary, and in recent years also in Spain [5, 9, 10]. However, to our knowledge, detection of PToV in China has not been reported. In 2011 winter, there were epidemic outbreaks of diarrhea that occurred with high morbidity and mortality in China, which has caused great economic losses. Diarrhea samples were collected for examination of enteric pathogens, in which PToV was included. In this study, we reported the first detection of PToV in southwest China and analyzed the phylogenetic relationships between the Chinese PToV and PToV reference strains as well as other representative toroviruses. A survey for other enteric pathogens was also conducted and statistical analysis of the epidemiological study with regard to clinical signs (diarrhea) was performed to reveal any association of PToV infection with diarrhea in piglets.

## 2. Materials and Methods

### 2.1. Specimens Collection

168 samples of feces or intestines from piglets that died of severe diarrhea from 20 farms in southwest China were collected during the winter of 2011, when there were epidemic outbreaks of diarrhea that occurred with high morbidity and mortality, which has caused great economic losses. Of note, most of the sampled piglets were one to three weeks old, and antibiotic treatment was invalid in all sampled piglets.

### 2.2. RNA Extraction and RT-PCR

For viruses detection, intestinal mucosa was grinded with liquid nitrogen and then mixed with intestinal contents. The mixture was diluted in 2 × volume (wt/vol) of phosphate-buffered saline (PBS, pH 7.4) and finally clarified by low-speed centrifugation at 3,000 ×g for 10 min. The supernatants were subsequently collected and subjected to RNA extraction. Total RNA of supernatants were extracted using TaKaRa RNAiso Reagent and dissolved in DEPC treated water, respectively. The strand cDNA was synthesized by reverse transcriptase (RT) using TaKaRa reverse transcription system. A primer pair targeting the spike (S) protein gene was used for the detection of PToV. The PCR was performed at 94°C for 5 min, followed by 30 cycles of 94°C for 30 s, 50°C for 30 s, and 72°C for 30 s, followed by a final extension at 72°C for 7 min. All specimens were also tested for the presence of PEDV (porcine epidemic diarrhea coronavirus), PRV A–C (porcine group A–C rotaviruses), TGEV (transmissible gastroenteritis coronavirus), AV (astroviruses), MRV (mammalian orthoreovirus), calicivirus (PSaV (porcine sapovirus), and PNoV (porcine norovirus) in accordance with the methods in previous studies [11–16] and PKBV (porcine kobuvirus) by the RT-PCR method established by our own laboratory and the PCR program was performed as described above with slight modifications. The used PCR specific primers were listed in Table 1.

### 2.3. Cloning of cDNA and Sequencing

To confirm the specificity of the fragments obtained by RT-PCR, PCR products were purified and used for sequencing. The purposed band, about 450 bp, was excised and then purified using Biomed gel extraction kit (BEIJING BILOMED CO., LTD) according to the manufacturer's instructions. The resulting products were cloned into pMDT-19 simple vector (Takara) for sequencing.

### 2.4. Sequence Analysis

The nucleotide and deduced amino acid sequences of the partial S gene were compared with other known toroviruses on the GenBank. Sequence similarity analysis was performed for the aligned nucleotide (excluding primer pair sequences) and amino acid sequences by the Clustal W method using the Megalign 7.2 program of Lasergene software (DNASTAR, Madison, WI, USA). Phylogenetic trees were carried out based on nucleotide alignments using the MEGA 5 program.

## 3. Results and Discussion

Using the RT-PCR assay (targeting a 451 bp fragment of the S gene of PToV), 9 out of 20 farms were positive for PToV. Among the 9 farms, two farms tested positive for PToV alone, while the remaining 7 farms had mixed infection with other viruses tested; no consistent association between PoTV and these viruses was observed. For other tested enteric pathogens, PEDV, PKBV, and PRV A had high positive rates which were 55% (11 out of 20 farms), 70% (14 out of 20 farms) and 75% (15 out of 20 farms), respectively, while none of the samples were positive for PRV B and calicivirus (PSaV and PNoV). Besides, 40% (8 out of 20 farms) samples were MRV positive, and positive rates of AV and TGEV were both 25% (5 out of 20 farms). Summary of enteric pathogens present in the porcine samples obtained from diarrheic pigs was listed in Table 2.

The newly determined sequences have been deposited in the NCBI nucleotide sequence database and assigned the following accession numbers: KC340952 (farm numbers 1 and 2); KC340953 (farm number 7); KC340954 (farm numbers 8 and 11); KC340955 (farm number 10); KC340956 (farm number 13); KC340957 (farm number 14); KC340958 (farm number 18).

**Figure 1:**
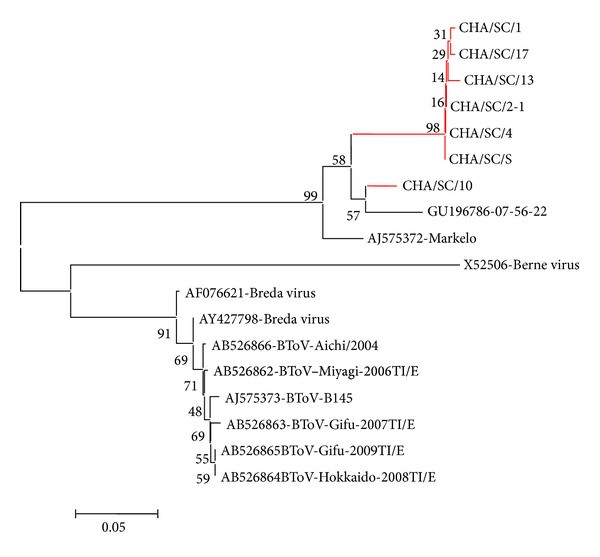
Phylogenetic analysis of partial PToV-S gene sequences obtained from Chinese farms and torovirus sequences available in GenBank. Gene sequences were aligned using the ClustalW method and the phylogenetic tree was performed by the neighbour joining method using 1000 bootstrap. Sequences from this study were marked red.

For 9 farms positive for PToV, the RT-PCR yielded a product of the anticipated size of 451 bp. Sequence analysis confirmed that the product was porcine torovirus specific. Those that shared the same sequence were neglected. Pairwise comparison of nucleotide sequences of the partial S gene confirmed that the strains are more closely related to the porcine torovirus. Comparison of the nucleotide (exclude the primer sequences) and deduced amino acid sequences of the fragment of the S gene of Chinese PToV strains showed that the Chinese PToV strains were highly conserved for the region, which had 90.2%–99.8% nucleotide and 93.7%–99.3 deduced amino acid identity with each other, and they formed a single lineage on the phylogenetic tree (Figure 1). The Chinese PToV strains were 90.9%–95.1% and 89.4%–96.5% identical to those porcine torovirus (AJ575372.1; GU196786.1) while only 38.3%–40.7% and 8.7%–10.8% to those of bovine torovirus. While, among the Chinese PToV strains, 6 strains clustered most closely with the the PToV Markelo/Netherlands strain, KC340955 clustered with GU196786. These results indicated that different PToV strains were circulating in China. Previous researches suggested that more than two different PToV strains could circulate simultaneously in an area; moreover individual animals could be infected by two strains during their productive life [5, 17].

In China, PToV-associated diarrhea has not been reported; even there was little information about PToV epidemiology. However, longitudinal, serological, and virological studies on PToV in piglets were carried out in Spanish and Europe and also RT-PCR method and real-time PCR were developed to detect PToV qualitatively or quantitatively in Korea [5, 6, 8, 17–19]. PToV epidemiology in China should be paid attention to. Our study first reported the existence of PToV in China, and PToV molecular epidemiology was also conducted in the study.

PToV has been detected in swine diarrhea samples and also high incidence of PToV infection in diarrhea samples was observed in our study. It was worth noting that two diarrhea samples were tested positive for PToV alone, and a previous study has reported a diarrhea sample tested positive for PToV alone when a survey for enteric pathogens in diarrheic pigs was carried out [20]. However, the impossibility of growing the virus in culture cells has precluded the development of PToV. We could not make the conclusion if there was any necessary connection between the two, or a relationship exists between PoTV and the other enteric pathogens identified. We could not ignore the importance of PToV and diagnosis of porcine diarrhea should include PToV examination. Further studies to reveal the epidemiological status of PToV infection in China are needed to be developed. In addition to investigating the molecular epidemics using RT-PCR, further immunological method should be established to detect serological prevelence of porcine torovirus in Chinese swine herds. Future researches will focus on the epidemiology and pathogenic potential of PoTV.

## Figures and Tables

**Table 1:** RT-PCR and nested PCR primers used in this study.

Target viruses^a^	Target genes^b^	Primer sequence, 5′–3^′c^	Size (bp)	Source or reference
PToV	S	F: ACCCCTGCCTGAGGTTTCYTT R: AGCACGACGTTGTCTRCGTGT	451	Established by our own laboratory
PEDV	S	F: TTCTGAGTCACGAACAGCCA R: CATATGCAGCCTGCTCTGAA	651	Kim et al. (2001) [11]
PKBV	3D	F: TGGATTACAAGTGTTTTGATGC R: TGTCGTAGAACTCCTTGATGAA	313	Established by our own laboratory
PRV A	VP6	F: AAAGATGCTAGGGACAAAATTG R: TTCAGATTGTGGAGCTATTCCA	308	Elschner et al. (2002) [12]
		nF: GACAAAATTGTCGAAGGCACATTATA nR: TCGGTAGATTACCAATTCCTCCAG	121	
PRV B	NSP2	F: CTATTCAGTGTGTCGTGAGAGG R: GCAGACAAGCTAGCCCGCTTCG	434	Gouvea et al. (1991) [13]
PRV C	VP6	F: CTCGATGCTACTACAGAATCAG R: AGCCACATAGTTCACATTTCATCC	366	Gouvea et al. (1991) [13]
		nF: CTCGATGCTACTACAGAATCAG nR: GGGATCATCCACGTCATGCGT	328	
TGEV	ORF1b	F: GGGTAAGTTGCTCATTAGAAATAATGG R: CTTCTTCAAAGCTAGGGACTG	1006	Kim et al. (2010) [14]
	S			
AV	RdRp	F11: GARTTYGATTGGRCKCGKTAYGA F12: GARTTYGATTGGRCKAGGTAYGA F21: CGKTAYGATGGKACKATHC F22: AGGTAYGATGGKACKATHC R1: GGYTTKACCCACATICCRAA	422	Chu et al. (2008) [15]
MRV	L1	F: GCATCCATTGTAAATGACGAGTCTG R: CTTGAGATTAGCTCTAGCATCTTCTG	416	Decaro et al. (2005) [16]
		nF: GCTAGGCCGATATCGGGAATGCAG nR: GTCTCACTATTCACCTTACCAGCAG	344	
PSaV and PNoV	RdRp	F: GATTACTCCAAGTGGGACTCCAC R: TGACAATGTAATATCACCATA	319	Kim et al. (2010) [14]

^a^PEDV: porcine epidemic diarrhea coronavirus; PKBV: porcine kobuvirus; PRV A–C: porcine group A–C rotaviruses; TGEV: transmissible gastroenteritis coronavirus; AV: astroviruses; MRV: mammalian orthoreovirus; PSaV: porcine sapovirus; PNoV: porcine norovirus.

^b^ORF: S: spike protein; 3D: RNA dependent RNA polymerase; VP6: viral protein 6; NSP2: nonstructural protein 2; ORF1b: open reading frame 1b; L1: large segment 1; RdRp: RNA dependent RNA polymerase.

^c^F: upstream primer for RT-PCR; R: downstream primer for RT-PCR; nF: upstream primer for nested PCR; nR: downstream primer for nested PCR.

**Table 2:** Summary of enteric pathogens present in piglets with diarrheic obtained from 20 farms.

Farm no.	PToV	PEDV	PKBV	PRV A	PRV B	PRV C	TGEV	AV	MRV	PSaV and PNoV
1	+	+	−	+	−	−	+	−	−	−
2	+	+	+	+	−	−	−	−	−	−
3	−	−	+	+	−	+	−	−	+	−
4	−	+	+	+	−	−	+	−	−	−
5	−	+	−	+	−	−	−	−	+	−
6	−	+	+	+	−	−	−	+	+	−
7	+	−	−	−	−	−	−	−	−	−
8	+	−	+	−	−	−	−	−	−	−
9	−	−	−	+	−	+	−	−	−	−
10	+	+	+	+	−	−	+	−	−	−
11	+	+	+	−	−	−	−	+	+	−
12	−	−	+	−	−	−	−	+	+	−
13	+	−	+	+	−	−	−	−	+	−
14	+	−	+	+	−	−	+	−	−	−
15	−	+	+	+	−	−	−	−	+	−
16	−	+	+	+	−	−	−	−	+	−
17	−	+	−	+	−	−	−	+	−	−
18	+	−	−	−	−	−	−	−	−	−
19	−	+	+	+	−	−	+	−	−	−
20	−	−	+	+	−	−	−	+	−	−

(+) indicates a positive result in PCR; (−) indicates a negative result in PCR.
